# Phosformer: an explainable transformer model for protein kinase-specific phosphorylation predictions

**DOI:** 10.1093/bioinformatics/btad046

**Published:** 2023-01-24

**Authors:** Zhongliang Zhou, Wayland Yeung, Nathan Gravel, Mariah Salcedo, Saber Soleymani, Sheng Li, Natarajan Kannan

**Affiliations:** School of Computing, University of Georgia, GA 30602, USA; Institute of Bioinformatics, University of Georgia, GA 30602, USA; Institute of Bioinformatics, University of Georgia, GA 30602, USA; Department of Biochemistry and Molecular Biology, University of Georgia, GA 30602, USA; School of Computing, University of Georgia, GA 30602, USA; School of Data Science, University of Virginia, VA 22903, USA; Institute of Bioinformatics, University of Georgia, GA 30602, USA; Department of Biochemistry and Molecular Biology, University of Georgia, GA 30602, USA

## Abstract

**Motivation:**

The human genome encodes over 500 distinct protein kinases which regulate nearly all cellular processes by the specific phosphorylation of protein substrates. While advances in mass spectrometry and proteomics studies have identified thousands of phosphorylation sites across species, information on the specific kinases that phosphorylate these sites is currently lacking for the vast majority of phosphosites. Recently, there has been a major focus on the development of computational models for predicting kinase–substrate associations. However, most current models only allow predictions on a subset of well-studied kinases. Furthermore, the utilization of hand-curated features and imbalances in training and testing datasets pose unique challenges in the development of accurate predictive models for kinase-specific phosphorylation prediction. Motivated by the recent development of universal protein language models which automatically generate context-aware features from primary sequence information, we sought to develop a unified framework for kinase-specific phosphosite prediction, allowing for greater investigative utility and enabling substrate predictions at the whole kinome level.

**Results:**

We present a deep learning model for kinase-specific phosphosite prediction, termed Phosformer, which predicts the probability of phosphorylation given an arbitrary pair of unaligned kinase and substrate peptide sequences. We demonstrate that Phosformer implicitly learns evolutionary and functional features during training, removing the need for feature curation and engineering. Further analyses reveal that Phosformer also learns substrate specificity motifs and is able to distinguish between functionally distinct kinase families. Benchmarks indicate that Phosformer exhibits significant improvements compared to the state-of-the-art models, while also presenting a more generalized, unified, and interpretable predictive framework.

**Availability and implementation:**

Code and data are available at https://github.com/esbgkannan/phosformer.

**Supplementary information:**

Supplementary data are available at *Bioinformatics* online.

## 1 Introduction

Protein phosphorylation is an important post-translational modification (PTM), catalyzed by protein kinase enzymes (Manning *et al.*, 2002). As one of the most widely observed and well-studied PTMs, phosphorylation is used as a means of cellular signaling which depends on the right kinase phosphorylating the right substrate at the right place and time (Ma *et al.*, 2005; Pawson and Scott, 1997). Consequently, misregulation may lead to a variety of diseases such as cancer, diabetes, and developmental defects (Viatour *et al.*, 2005). Although advances in mass-spectrometry and antibody assays have allowed the high throughput discovery of phosphosites across proteomes (Aslebagh *et al.*, 2019; Ramazi and Zahiri, 2021), identifying the kinases that phosphorylate specific serine/threonine or tyrosine residues is a major challenge and relies on low-throughput antibody-based assays focused on specific kinases and substrates (Elia *et al.*, 2003; Songyang and Cantley, 1998). Consequently, a vast majority of the phospho-proteome remains poorly annotated (Needham *et al.*, 2019) and the lack of kinase labels for phosphosites presents a major bottleneck in understanding cellular signaling networks regulated by the nearly 500 protein kinases encoded in the human genome.

Due to the cost and time associated with the experimental characterization of kinase–substrate interactions, much research has gone toward building machine learning models for predicting phosphorylation. Early models were aimed toward general phosphosite prediction (Wang *et al.*, 2017, 2019), while more recent models have improved resolution—predicting kinase-specific phosphosites (Kirchoff and Gomez, 2022; Luo *et al.*, 2019; Wang *et al.*, 2019). However, the latter task has been challenging as much of the kinase-specific phosphorylation sites have been skewed towards a subset of well-studied kinases. In order to develop a more accurate model for kinase-specific phosphosite prediction, we take a domain knowledge-driven approach. Here, we first systematically highlight important biological intuitions relating to protein phosphorylation, review how each biological intuition is modeled by existing machine learning approaches, and propose ways in which these approaches may be improved.

Phosphorylation is highly dependent on the sequence, structure, and other contextual features such as cellular localization and scaffolding of both the kinase and the substrate proteins. Consequently, many prediction models have demonstrated improved performance by utilizing sequence (Zhou *et al.*, 2004), structural (Dou *et al.*, 2017), and functional features (Fan *et al.*, 2014; Kuleshov *et al.*, 2021; Yang *et al.*, 2021). Furthermore, some models also utilize evolutionary features (Lai *et al.*, 2012), which are useful heuristics for identifying closely related proteins with similar sequences, structures, and functions. While explicitly including these features can improve performance, a protein’s primary sequence should theoretically already encode structural, functional, and evolutionary information. In fact, protein language models (pLMs) have been shown to be capable of implicitly extracting this information in an unsupervised manner (Rives *et al.*, 2021). Using pLMs, it should be possible to train a prediction model which only requires primary sequence information. This would avoid the need to develop an independent feature generation pipeline as well as avoid potential human biases in feature selection and engineering.

All protein kinases perform the same reaction, however different kinase families recognize and catalyze different substrate sequence motifs (Johnson *et al.*, 2023). In order to segregate kinase families with different substrate specificities, a common approach is to train multiple models—each specialized towards a specific kinase family or group (Luo *et al.*, 2019; Wang *et al.*, 2017; Yang *et al.*, 2021). Although this can improve prediction accuracy, training multiple models further divide the limited training data and makes it harder for a single model to learn general features about phosphorylation. Towards developing a more unified framework, recent work has proposed a single model that works on five kinase families (Kirchoff and Gomez, 2022). Taking this one step further, a framework that is capable of representing any kinase–substrate pair would allow for even greater investigative utility and the potential to extend the predictive model toward understudied protein kinases.

Negative examples, sequences that are not phosphorylated or not phosphorylated by a specific kinase, are another important aspect of training machine learning models. Defining negative examples for general phosphosite prediction is relatively straightforward—all serine, threonine, or tyrosine residues that lack evidence of phosphorylation. These may include buried residues on substrate sequences that cannot be accessed by the kinase. In comparison, kinase-specific negative examples are more difficult to define due to the paucity of experimentally validated negative datasets. Thus, most methods for kinase-specific phosphosite prediction simply define negative examples as residues with no evidence of phosphorylation (Luo *et al.*, 2019; Wang *et al.*, 2017; Yang *et al.*, 2021). However, this definition of negative examples arbitrarily introduces random peptides that are very easy to distinguish from true phosphosites, resulting in inflated prediction performance. Furthermore, some models are trained using a balanced ratio of positive to negative examples (Luo *et al.*, 2019; Yang *et al.*, 2021), whereas phosphorylatable sequence motifs actually make up a tiny fraction of biological sequence space. Improved curation of negative examples would allow predictive models to distinguish the boundary separating phosphorylatable and non-phosphorylatable residues hence accelerating the training for kinase-specific phosphorylation prediction.

Here, we implemented the proposed improvements towards developing Phosformer, a deep learning model which makes kinase-specific phosphosite predictions based on a pair of inputs—the unaligned kinase domain sequence and the substrate peptide sequence. Using a two-step training procedure, we first pre-trained Phosformer to understand the ‘biological language’ from unlabeled protein sequences, then fine-tuned the model to predict kinase-specific phosphosites. Despite only being trained on primary sequences, we demonstrate that Phosformer learns to distinguish kinase evolutionary groups as well as functionally similar kinases that recognize similar substrate motifs. In order to fine-tune the model, we curated a dataset that defines different types of negative examples and developed a novel multi-level negative sampling strategy. Furthermore, we also implemented a means of training with extreme class imbalance. Our benchmarks indicate that Phosformer exhibits significant improvements compared to other state-of-the-art models, while also presenting a more generalizable and unified predictive framework.

## 2 Materials and methods

### 2.1 Dataset curation and split

We manually curated a kinase-specific phosphosite dataset by integrating multiple data sources. Each row in the dataset denotes an unaligned kinase domain sequence, an 11-mer peptide sequence, and a positive/negative label indicating whether the kinase phosphorylates the middle residue of the peptide sequence. For all peptides in our dataset, the middle residue is always serine, threonine, or tyrosine. If the phosphosite occurs within five residues of the N or C-terminus of the protein, the sequence is padded by a special token to ensure that all peptides have equal length and that the phosphosite occurs in the center position.

**Fig. 1.**
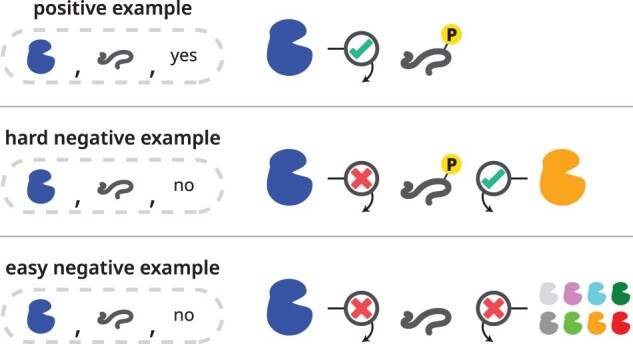
Definition of kinase-specific phosphorylation dataset. For each kinase, positive examples are defined as substrates that contain center S/T/Y sites that are known to be phosphorylated by this given kinase. The hard negative examples are defined as substrate that contains center S/T/Y sites that are known to be phosphorylated by other kinases but not the paired kinase. The easy negative examples are defined as substrate that contains center S/T/Y sites that are not known to be phosphorylated by any kinase

- **Positive examples**: Experimentally validated kinase-specific substrates were curated from three publicly accessible databases: Phospho.ELM (Dinkel *et al.*, 2011), PhosphoNetwork (Hu *et al.*, 2014), and PhosphoSitePlus (Hornbeck *et al.*, 2012). While consolidating these databases, we verified each data point by cross-referencing the UniProt Database (UniProt Consortium, 2020). Overall, we curated 24 534 unique kinase–substrate pairs spanning 800 unique kinases from 13 organisms.
- **Negative examples**: Defining a negative example is innately challenging as phosphosite databases only report positive results, while negative examples must be inferred. In the context of our dataset, we refer to **positive examples** as kinase-specific phosphosites. For every kinase, we explicitly define two separate categories of negative examples (Fig. 1):
  - **Hard negative examples** which are peptides that have experimental evidence of phosphorylation but not by the paired kinase.
  - **Easy negative examples** which are random peptides containing S/T/Y center residue with no evidence of being phosphorylated by any kinase.
    Negative examples were generated by using all unique 11-mer peptide fragments that appear within the full-length substrate proteins in our dataset. Although many previous methods used easy negative examples in their model training (Luo *et al.*, 2019; Wang *et al.*, 2017; Yang *et al.*, 2021), we argue that those random S/T/Y sites are very easy to distinguish. To facilitate fair and meaningful comparison, we decide not to include easy negative examples in the AUC ROC and AUC PRC score evaluation to avoid inflated performance.
- **Dataset split**: We split our positive examples into non-overlapping training, validation, and testing sets at a 70:15:15 ratio for kinases with more than 50 positive examples, while kinases with less than 50 positive examples were limited to the training dataset. A total of 106 unique kinases were included in the testing and validation sets. For each positive example in the training set, we added (n+m) negative examples (*n* easy negative examples, *m* hard negative examples), where *n* and *m* is a tunable parameter (see Section 2.2). We also developed multiple data augmentation techniques applied to training data to make the model more robust (see Section 2.2). For each positive example in the validation and testing sets, we added one hard negative sample. We defined an additional negative testing set consisting of 8504 easy examples for benchmarking purposes. Due to the highly unbalanced ratio of positive to negative examples, the majority of negative examples were not used. The validation dataset is used for hyperparameter tuning while the testing dataset is held out as a standard benchmark dataset for comparing all models.

**Fig. 2.**
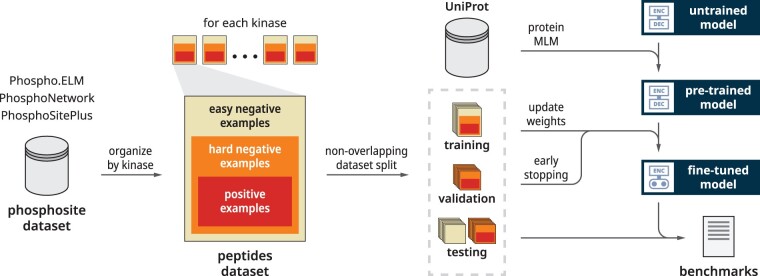
General overview of our complete end-to-end workflow. Starting from the left, we curated a dataset of kinase–peptide pairs from three publicly accessible phosphosite databases (Dinkel *et al.*, 2011; Hornbeck *et al.*, 2012; Hu *et al.*, 2014). Data points were grouped by kinase, each of which is associated with a number of peptides that can be further classified as easy negative examples, hard negative examples, and positive examples. Data points were split into a non-overlapping training set, a validation set, and two separate testing sets. Our deep learning model was first pre-trained by MLM. The pre-trained model was fine-tuned for kinase-specific phosphosite prediction using the training and validation sets. The final model was benchmarked using the two testing datasets with other available methods

### 2.2 Protein-specific data augmentation

Data augmentation techniques help produce more robust models and avoid overfitting by increasing the training set size via meaningful modifications to the original data. Here, we developed four augmentation strategies that are compatible with our protein sequence inputs (Taujale *et al.*, 2021). Our best-performing model was trained using a combination of all four augmentation strategies. These benchmarks are later shown in the results.

- **Resampling under-represented kinases**: Although our curated dataset includes examples from 800 unique kinases, the number of experimentally validated substrates peptides per kinase is highly skewed. For instance, PKA-α has 1118 positive examples, the most in our dataset, while 122 kinases have only one positive example each. To prevent the model from overfitting kinases with more training examples, we randomly resampled positive examples for kinases with fewer training examples in order to balance the training data. Kinases with more than 50 positive examples (included in the testing set) were upsampled to 1118, while kinases with less than 50 positive examples (only included for training) were upsampled to 50. To avoid repeatedly using the exact same examples, we also implement several permutation-based data augmentation strategies which are described in the following sections. This introduces variation and ensures that resampled examples are different.
- **Shifting kinase domain boundaries**: In contrast to most phosphosite prediction models which encode specific kinases by separated models, Phosformer encodes kinases solely based on their unaligned kinase domain sequence. Consequently, the same kinase could be represented by slightly different sequences depending on how the kinase domain boundaries are defined. In order to make the model robust to this variability, we randomly shift both the N- and C-terminal boundaries of the kinase domain by up to 5 residue positions. Shifts are performed independently at each terminus. This augmentation also prevents the model from relying on precise domain boundaries as a means for identifying the kinase.
- **Kinase sequence masking**: We added noise to sequence information by randomly replacing 5% of kinase sequence tokens with the <mask> special token which indicates that the identity of the amino acid is uncertain. This is conceptually similar to a common augmentation in computer vision research, adding random Gaussian noise to images, which aims to create variations in the input data without changing the contextual meaning. Furthermore, this augmentation also disallows the model from over-relying on a small subset of highly informative residue positions and forces the model to consider the entire kinase sequence.
- **Multilevel negative sampling**: As previously described under data curation, we define two categories of negative examples. The easier of the two categories is easy negative examples—peptides that contain an S, T, or Y residue in the center but have no evidence of being phosphorylated by any kinase. These may consist of buried sequence regions that would be inaccessible to the kinase. The more difficult category is hard negative examples—peptides that are known to be phosphorylated but for which kinase labels are not the paired one. For instance, the hard negative example could include a peptide–kinase pair in which a basophilic kinase is paired with the substrate of a proline-directed kinase. We design a novel multilevel negative sampling strategy by including (n+m) negative examples (*n* easy negative examples, *m* hard negative examples) for each positive example in the training dataset, where *n* and *m* is a tunable parameter. A higher n value corresponds to a more severe class imbalance. These negative examples can also undergo kinase shifting and masking augmentations, while resampling augmentation was not required due to the abundance of negative examples (Fig. 2).

### 2.3 Phosformer model training

**Fig. 3.**
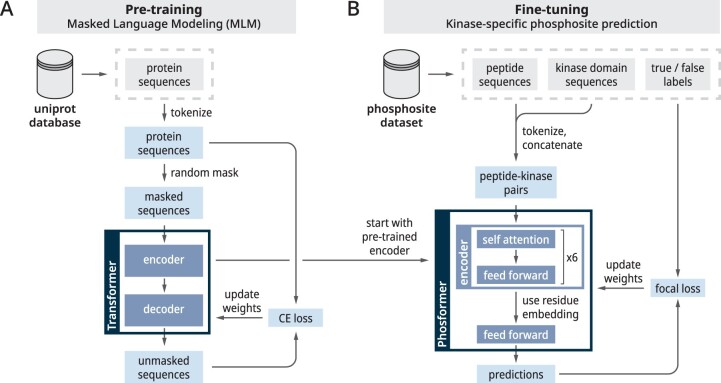
General overview of our model training workflow. (**A**) We pre-train a Transformer model on a MLM on full-length protein sequences from UniProt. Our model was trained by cross-entropy (CE) loss. (**B**) After pre-training, we fine-tune our model to predict whether a given kinase phosphorylates a given peptide sequence. The fine-tuning stage uses focal loss to offset the large imbalance ratio

We trained the Phosformer model in two phases. In the pre-training phase (Fig. 3A), the model was taught to understand the ‘language of life’ by masked language modeling (MLM) on biologically observed protein sequences. In the fine-tuning phase (Fig. 3B), the model was taught to predict kinase-specific phosphorylation. Specifically, the model takes two inputs—an 11-mer peptide sequence and an unaligned kinase domain sequence. Based on the inputs, Phosformer predicts the likelihood of the middle phosphorylatable residues (S/T/Y) in the peptide to be phosphorylated by the given kinase.

To provide the rationale for our design, we aimed to define a universal framework that allows us to model the relationship between any protein kinase to any potential substrate peptide. For this reason, we explicitly encode protein kinases using their primary sequence rather than separating them into different models which would limit the model’s scope to a predefined set of kinases. In order to avoid potential biases relating to feature engineering, we do not provide our model with any additional features such as kinase evolutionary classifications, structure-functional information, and physico-biochemical properties. Furthermore, we reason that our specific model architecture should be capable of inferring this information based on the primary sequence alone.

#### 2.3.1 Protein language pre-training

We utilize a Transformer model consisting of an encoder and decoder where the encoder consists of 6 Attention layers. Each Attention layer consists of 12 attention heads and produces an output tensor containing 768 embedding dimensions. The Transformer model reads variably sized (unaligned) protein sequences as input. As pre-processing, protein sequences are encoded as a series of sequence tokens, each representing an amino acid. In addition to the sequence tokens, we also add two special tokens to denote the beginning and end of the protein sequence. The encoder reads tokenized sequences and returns sequence embedding vectors of a size (t+2768) where *t* is the sequence length. Performing the opposite function, the decoder reads sequence embedding vectors and returns tokenized sequences.

The model was trained using the masked language modeling (MLM) objective (Vaswani *et al.*, 2017) on a corpus of kinase and substrate sequences. Specifically, we used 295 320 diverse kinase domain sequences from the NCBI non-redundant database (retrieved July 13, 2021) spanning 18 832 unique organisms. We also used 5338 full-length substrate proteins, defined by all substrates which appear in our curated kinase-specific phosphosite dataset, from the UniProt database (retrieved January 23, 2022) spanning 29 unique organisms. In total, the kinase portion includes 86 944 788 amino acids, while the substrate portion includes 3 845 761 amino acids. To outline the MLM process, we randomly select 15% of amino acid tokens, replace them with mask tokens, then try to predict the identity of the original amino acid token based on the available context. This can be formalized as:

(1) $$\begin{array}{c} L_{\text{MLM}}=(X_{\Pi }|X_{-\Pi })=\frac{1}{K}\sum_{k=1}^{K}\;\text{log}\;p(X_{\Pi_{k}}|X_{-\Pi };\theta ) \end{array},$$

where $X_{\Pi }$ is the set of masked tokens in the input, $X_{-\Pi }$ is the set of unmasked tokens, *K* is the number of masked tokens in the input, and the indexes of the masked tokens in the input as $\Pi =v_{1},v_{2},v_{3},\ldots ,v_{K}$. The cross-entropy loss was calculated for back propagation during training. The pre-training step uses the AdamW optimizer with a learning rate of 5e−5. The model was trained for 50 epochs on 8 NVIDIA A5000s with a batch size of 48 per device.

#### 2.3.2 Kinase-specific phosphorylation fine-tuning

We constructed the Phosformer model using parts of the pre-trained Transformer model, followed by a new module to facilitate phosphosite prediction. The Phosformer architecture consists of the pre-trained encoder consisting of six Attention layers, followed by a feed-forward layer, ending with a binary classification layer that corresponds to a positive or negative prediction. Prediction values were converted into probabilities using the softmax function. To summarize the input and output, Phosformer reads sequence information as input and provides a probability value as output.

In order to train Phosformer, we modeled kinase-specific phosphosite prediction as a context-based question-answering (CBQA) task, a training objective inspired from natural language processing (Yoon *et al.*, 2019). To outline the CBQA system, the model is given a ‘context’ and ‘question’ to which it must provide an ‘answer’. Within our model, the peptide is modeled as the ‘context’, the kinase sequence is modeled as the ‘question’, and the probability of phosphorylation is modeled as the ‘answer’.

During pre-processing, the peptide and kinase sequences are tokenized, then concatenated to form a single peptide–kinase pair. Specifically, this peptide–kinase pair consists of the tokenized 11-mer peptide sequence flanked by two special tokens to denote the start and end, followed by the tokenized kinase domain sequence which is also flanked by two special tokens. Dash characters, used to pad peptide sequences, were encoded using the <pad> special token. The encoder reads the tokenized peptide–kinase pair and returns an embedding of size $\left(t_{\text{peptide}}+t_{\text{kinase}}+4768\right)$. From this embedding, the position corresponds to the middle residue of the phosphate—the potential phosphosite—is directed into a feed-forward layer which is followed by the final binary prediction.

Our training dataset contains significantly more negative examples than positive examples. In order to effectively train the model, we sought a loss function that is capable of dealing with the class imbalance and preventing the model from solely focusing on negative examples. To this end, we implement the focal loss function (Lin *et al.*, 2017), a dynamically scaled cross-entropy loss function that focuses on learning commonly misclassified examples. This is formalized as:

$$\begin{array}{c} \text{Focal}\;\text{Loss}(p_{t})={\left(1-p_{t}\right)}^{\gamma }\;\text{log}(p_{t}) \end{array},$$

where $p_{t}$ is the predicted probability and γ is the dynamic scaling factor. Setting the dynamic scaling factor to a larger value effectively down-weights the contribution from easily classified examples during training so as to put more focus on the difficult examples. The fine-tuning step used the AdamW optimizer with a learning rate of 2e-5, focal loss scaling factor as 2 and the early stopping steps as 5.

### 2.4 Performance evaluation

We utilize several metrics for evaluating the performance of our model in kinase-specific phosphosite prediction. First, we used the Area Under Curve Receiver Operating Characteristic (AUC ROC) score, a commonly used metric reported in previous studies (Kirchoff and Gomez, 2022; Luo *et al.*, 2019; Wang *et al.*, 2017; Yang *et al.*, 2021):

(2) $$\begin{array}{c} \text{AUC}-\text{ROC}=\int_{0}^{1}T\text{PR}({\text{FPR}}^{-1}(x))dx \end{array}.$$

Since our curated dataset includes significantly more negative examples than positive examples, traditional metrics might provide inaccurate comparison under such circumstances. In order to account for this class imbalance and provide a fair comparison, we used the Area Under Curve Precision-Recall Curve (AUC PRC) score (Saito and Rehmsmeier, 2015).

(3) $$\begin{array}{c} \text{AUC}-\text{PRC}=\int_{0}^{1}P\text{recision}({\text{Recall}}^{-1}(x))dx \end{array}.$$

Finally, we measure our model’s susceptibility to false-positive predictions by quantifying the false positive rate (FPR) which evaluates the model’s performance given a dataset with only negative examples.

(4) $$\begin{array}{c} \text{FPR}=\text{FP}/(\text{TN}+\text{FP}) \end{array}.$$

TP, TN, FP and FN stand for True Positive, True Negative, False Positive, and False Negative respectively. TPR is defined by TP/(TP + FN), while FPR is defined by 1−TN/(TN + FP). Precision is defined by TP/(TP + FP) and Recall is defined by TP/(TP + FN). Higher AUC ROC and AUC PRC scores indicate better models, while lower FPR indicates that the model is less likely to generate false-positive predictions.

## 3 Results

### 3.1 Evaluation of data augmentation strategy

In order to find an optimal model for predicting kinase-specific phosphosites, we trained Phosformer with varying hyperparameters using the training and evaluation datasets. Afterward, we benchmarked these models using two different testing sets, both containing examples which were never seen during training. The first testing set only contained peptides that are known to be phosphorylated where half of them are paired with the correct phosphorylating kinase (positive examples) and half of them are paired with the incorrect kinase (hard negative examples). The second testing set only contained peptides with no evidence of phosphorylation where each example was paired with a random kinase (easy negative examples). We chose to define separate testing sets in order to independently benchmark different aspects of our model.

**Fig. 4.**
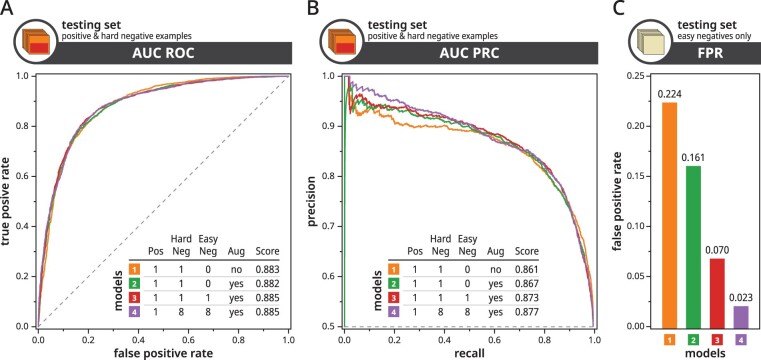
Comparison of model performance. We show AUC ROC (**A**), AUC PRC (**B**), and FPR (**C**) plots for four models trained with varying ratios of positive to negative examples, with(out) augmentations. We separate the models based on used training dataset and augmentation strategy. Within the legend table, each row corresponding to a trained model, The Pos column defines the positive data ratio, the HardNeg column defines the ratio of hard negative examples, and the EasyNeg column defines the ratio of easy negative examples. The Aug column defines whether to use the Mask+Shift+Resample augmentation strategy. As indicated by the icon on the top-left of each graph, the AUC ROC and AUC PRC plots were generated using the testing set containing a mix of positive and hard negative examples, while the FPR plot was generated using the separate testing set containing only easy negative examples

Our benchmarks indicate that our kinase-focused resampling, shifting, and masking augmentations improved the model’s ability to distinguish between kinase-specific substrates peptides based on AUC ROC and AUC PRC scores (Fig. 4A and B) and also improved the model’s ability to discriminate against non-phosphosites based on the FPR score (Fig. 4C). From a practical standpoint, these augmentations also make the model more robust to potential variations in the user-defined kinase domain sequences (Supplementary Fig. S3).

Next, we benchmarked the effects of our multilevel negative sampling strategy by introducing peptides with no evidence of phosphorylation into the training dataset. This dramatically improved the model’s ability to discriminate against non-phosphosites (Fig. 4C), also resulting in marginal improvements in kinase-specific phosphosite prediction (Fig. 4A and B). We gained further improvements by introducing even more negative examples in our training dataset—modeling the fact that phosphosite motifs represent a very small fraction of sequence space in biology. In order to effectively train these models, we used the focal loss (Lin *et al.*, 2017) which is capable of tolerating our extremely imbalanced ratio of positive to negative examples (Supplementary Fig. S2). Based on our benchmarks, we selected the Phosformer model trained with all augmentations, using a training dataset containing a 1 to 16 ratio of positive to negative examples. Unless otherwise stated, we use this model as our final model to benchmark and discuss in the following sections.

### 3.2 Evaluation of kinase-specific phosphosite predictions

**Fig. 5.**
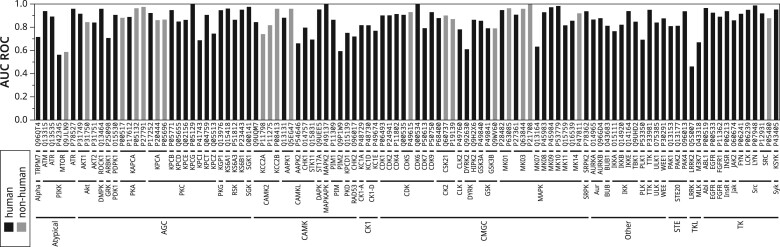
Model performance for diverse kinases. A bar chart depicts AUC ROC scores for the 106 kinases included in our testing set. Human kinases are denoted by black bars, while gray bars denote non-human kinases. The UniProt accessions of each kinase are provided across the *x*-axis. Under the accessions, we provide the kinase classification hierarchy which includes the gene, family, and group names

We further investigated the performance of Phosformer on individual kinases. Benchmarks indicate an AUC ROC score of 0.885 across all testing set examples (Fig. 4A) or a mean AUC ROC score of 0.860, averaged across all 106 kinases included in our testing set (Fig. 5). Phosformer offers relatively consistent performance with the majority of kinases scoring above 0.800. Furthermore, the model’s performance on kinases from different organisms is similar to their human orthologs. Overall results indicate that Phosformer is able to make accurate predictions across diverse families/groups and organisms. Given that the model was trained on sequence information alone, these results also indicate that our training curriculum allowed Phosformer to learn generalized features that are important for kinase-specific phosphosite prediction. Examples of these features are discussed in later sections.

### 3.3 Comparison with existing methods

We compared Phosformer against five recently published methods for phosphosite prediction using our two testing sets. Like the previous sections, AUC ROC and AUC PRC scores were calculated using the first testing set which contains both positive and hard negative examples, while FPR scores were calculated using the second testing set which contains only easy negative examples. Phosformer only utilizes the kinase domain and peptide sequences as input, thus we had to explicitly add additional features to satisfy the input requirements of other methods. These additional features included kinase family/group labels and protein–protein interactions. Many competing methods also implement multiple family/group-specific models which limit their generalizability. For instance, group-level models were not available for MusiteDeep (Wang *et al.*, 2017), while the TK and Src models were not accessible for DeepPhos (Luo *et al.*, 2019). Using a slightly different approach, EMBER is a single model that can accommodate inputs from five distinct kinase families (Kirchoff and Gomez, 2022). In comparison to these methods, Phosformer is a single model that can theoretically accommodate any input kinase, though predictions on understudied kinases not used in training should be interpreted with caution.

Phosformer makes predictions at the individual kinase level. Consequently, our model is not directly comparable to other methods which make predictions at the family/group level. In order to facilitate meaningful comparisons, we reported the performance of kinases organized by family/group labels. Compared to other models, Phosformer demonstrates superior performance in group-level predictions while demonstrating comparable or better performance across kinase families (Table 1). Among previously published models, our benchmarks reveal that group-level models tend to predict significantly more false positives than family-level models, as indicated by the FPR score. In comparison, Phosformer consistently exhibits low false-positive rates. Compared to other recently published methods, Phosformer allows predictions on more kinases, uses fewer features (requiring less user input), and exhibits state-of-the-art performance across the majority of protein kinome.

**Table 1.** We compare the performance of Phosformer to recently published methods for kinase-specific substrate phosphorylation prediction: MusiteDeep (Wang *et al.*, 2017), DeepPhos (Luo *et al.*, 2019), PhosIDN (Yang *et al.*, 2021), PhosIDNSeq (Yang *et al.*, 2021) and EMBER (Kirchoff and Gomez, 2022)

Level		MusiteDeep			DeepPhos			PhosIDN			PhosIDNSeq			EMBER			Phosformer		
Unified model		4 models			8 models			10 models			10 models			1 model			1 model		
Metric		AUC ROC	AUC PRC	FPR	AUC ROC	AUC PRC	FPR	AUC ROC	AUC PRC	FPR	AUC ROC	AUC PRC	FPR	AUC ROC	AUC PRC	FPR	AUC ROC	AUC PRC	FPR
Group	Atypical	–			0.809	0.834	0.177	0.686	0.7	0.146	0.797	0.783	0.392	–			**0.849**	**0.864**	**0.012**
	AGC	–			0.795	0.825	0.190	0.655	0.72	0.153	0.804	0.836	0.458	–			**0.898**	**0.900**	**0.018**
	CAMK	–			0.706	0.716	0.158	0.627	0.675	0.171	0.717	0.722	0.185	–			**0.830**	**0.813**	**0.009**
	CMGC	–			0.813	0.798	0.149	0.726	0.764	0.158	0.808	0.793	0.679	–			**0.898**	**0.882**	**0.006**
	TK	–			–			0.505	0.838	0.178	0.615	0.887	0.754	–			**0.931**	**0.910**	**0.049**
	CK1	–			–			–			–			–			**0.808**	**0.822**	**0.000**
	Other	–			–			–			–			–			**0.806**	**0.820**	**0.005**
	STE	–			–			–			–			–			**0.828**	**0.844**	**0.008**
	TKL	–			–			–			–			–			**0.605**	**0.612**	**0.011**
Family	PKC	0.826	0.851	0.101	0.756	0.78	0.046	0.649	0.712	0.159	0.809	0.821	0.209	0.687	0.096	0.516	**0.884**	**0.894**	**0.017**
	CDK	**0.923**	**0.915**	0.0416	0.897	0.874	0.024	0.859	0.867	0.062	0.891	0.857	0.074	0.789	0.181	**0.021**	0.907	0.884	0.026
	CK2	0.855	**0.891**	0.0865	0.852	0.862	0.035	0.676	0.714	0.131	0.848	0.839	0.122	0.841	0.226	**0.017**	**0.881**	0.884	0.031
	MAPK	0.911	0.903	0.0598	0.881	0.862	**0.016**	0.797	0.819	0.050	0.867	0.847	0.144	0.811	0.235	0.066	**0.924**	**0.915**	0.026
	Src	–			–			0.516	0.847	0.179	0.601	0.883	0.263	0.498	0.034	0.039	**0.927**	**0.907**	**0.031**
	Other families (38)	–			–			–			–			–			**0.857**	**0.856**	**0.019**

*Note*: Model performance was quantified by the average AUC-ROC score, AUC-PRC score and FPR score, stratified by kinase group and family. Empty fields indicate that the corresponding model is unable to make predictions for the corresponding kinase group or family. The model with the highest performance is highlighted with bold.

### 3.4 Phosformer learns evolutionary and biochemical features from primary sequences alone

Although phosphosite prediction typically requires protein sequence, structure, function and evolutionary features, Phosformer achieves state-of-the-art performance using just sequences. Further investigation reveals our novel training procedure allows Phosformer to implicitly learn biological features from unaligned primary sequences alone. The model was first pre-trained using MLM, then fine-tuned for kinase-specific phosphosite prediction. Starting from a randomly initialized model, the pre-training stage was designed to provide a general understanding of biologically observed protein sequences. In the fine-tuning stage, we encouraged the model to apply its existing knowledge toward phosphosite prediction. We then investigated the training process by comparing the pre-trained model against the fine-tuned model.

**Fig. 6.**
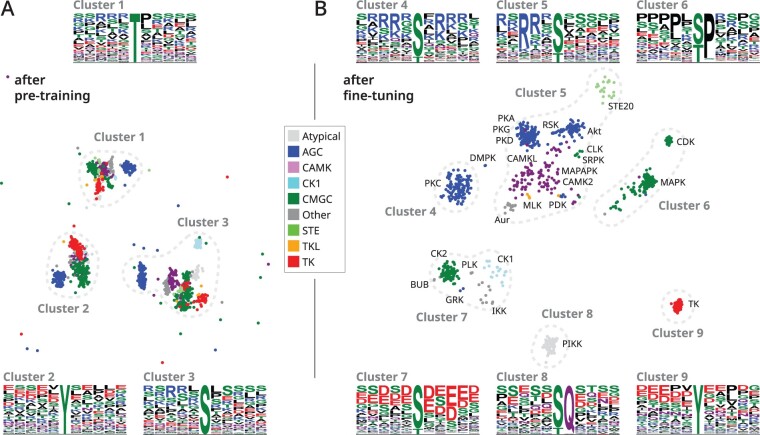
Comparison of embeddings vectors generated from the Phosformer model after two different training stages—(**A**) after pre-training and (**B**) after fine-tuning. Using our testing set containing peptide–kinase pairs, we generated sequence embeddings vectors using the encoder module. We plot UMAP projections of the residue embedding of the potential phosphosite, extracted from the full sequence embedding. Across both projections, we label nine major clusters defined by the boundaries in dotted lines. As previously stated, each data point represents an embedding vector of a specific peptide-kinase pair. The color of each data point corresponds to the kinase group as indicated by the legend, while more specific kinase family labels are directly provided on the scatter plot. We also show sequence logos for the 11-mer peptide sequences from each of the nine clusters

Our training curriculum directly affects how Phosformer interprets peptide-kinase sequence pairs. Using our testing dataset, we compared embedding vectors generated after pre-training to embeddings after fine-tuning. After pre-training, UMAP projections reveal that the model is able to organize peptides based on the identity of the potential phosphosite (Fig. 6A). Each of the three main clusters corresponds to serine, threonine, or tyrosine. Each of the main clusters further subdivides into smaller clusters corresponding to the major kinase evolutionary groups. In fact, previous studies have further shown that sequence embeddings can encode phylogenetic relationships (Yeung *et al.*, 2023b). Interestingly, data points that do not fall into the three main clusters correspond to peptides where the phosphosite is less than five residues away from the N or C-terminus. Overall results indicate that the pre-trained model understands general biochemical and evolutionary features pertaining to the peptide and kinase protein.

After fine-tuning, Phosformer learns to organize peptide-kinase sequence pairs based on substrate specificity. We generated embeddings from the fine-tuned model and created a UMAP projection which revealed six major clusters (Fig. 6B). Clusters 4 and 5 correspond to basophilic kinases, where cluster 4 more specifically prefers substrates containing basic residues on either side of the phosphosite, while cluster 5 prefers substrates containing basic residues preceding the phosphosite (Zhu *et al.*, 2005). Cluster 6 corresponds to proline-directed kinases (Kannan and Neuwald, 2004; Pinna and Ruzzene, 1996), cluster 7 to acidophilic kinases (Pinna and Ruzzene, 1996), cluster 8 to PIKK family kinases which recognize the SQ motif (Mordes and Cortez, 2008), and cluster 9 to tyrosine kinases (Hunter, 2009). Overall results indicate that the fine-tuned model has learned to recognize major substrate specificity groups—specialized knowledge on kinase function which builds off of the more general biological knowledge learned during pre-training.

**Fig. 7.**
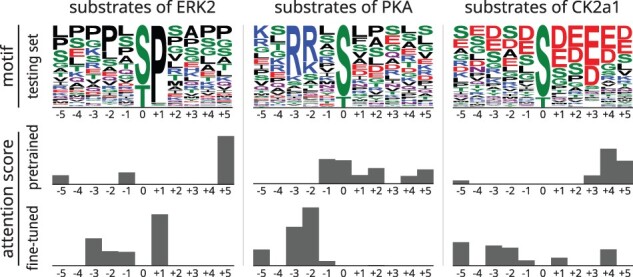
Substrate attention in the Phosformer model. On the top row, we show sequence logos for substrates of three kinases, taken from the testing set. The bar plots show the average attention scores for the Phosformer model after pre-training (upper) and after fine-tuning (lower)

Given that language models are also capable of learning protein functional motifs (Yeung *et al.*, 2023a), we also demonstrate Phosformer’s ability to recognize substrate specificity motifs by investigating which substrate residues the model is paying attention to. This is quantified as a function of the final Attention layer of the encoder (Supplementary Fig. S1). Using our testing set, a comparison between the pre-trained and the fine-tuned model indicates that Phosformer only learns to pay attention to substrate specificity determinants after fine-tuning. For example, given substrates of ERK2, the fine-tuned model directs the most attention to the P + 1 position which is a major determinant for proline-directed kinases (Pinna and Ruzzene, 1996), while the pre-trained model pays the most attention to the P + 5 position which is not a specificity determinant (Fig. 7 left). We observe similar results for PKA where the fine-tuned model directs the most attention to the P-2 and P-3 positions (Fig. 7 middle), which are major determinants for basophilic kinases (Zhu *et al.*, 2005). As for CK2a1, we note that the attention changes after fine-tuning, however, the attention is more equally distributed across the substrate sequence (Fig. 7 right), perhaps indicating a distributed contribution from all positions. Indeed, substrates of acidophilic kinases tend to be acidic residue patches (Pinna and Ruzzene, 1996). In addition to the canonical acidic residues, glutamate, and aspartate, we note that phosphorylatable residues such as serine can also become acidic after modification. This particular subset of acidophilic kinases is more specifically referred to as phosphate-directed kinases (Xu *et al.*, 1995). By directly analyzing the model’s ‘Attention’ mechanism, we find that our fine-tuning curriculum teaches Phosformer to recognize specificity determinants across diverse kinase substrate specificity groups.

## 4 Discussion

We present Phosformer, an interpretable deep learning model that archives a new state-of-the-art performance for kinase-specific phosphorylation predictions. Leveraging our novel Transformer-based architecture, Phosformer learns the ‘language of life’ in an unbiased and unsupervised fashion during pre-training. After fine-tuning using the kinase-specific phosphorylation dataset, Phosformer acquired biologically meaningful features that enable accurate predictions on substrates. Not requiring auxiliary features as input, the model is highly amenable to high-throughput predictions. The prediction pipeline is highly optimized to facilitate multi-threading or CUDA acceleration, enabling the model to make on average 220 predictions per second on a normal computer with a single RTX 2080 GPU card.

Phosformer represents major technical advantages for kinase-specific phosphorylation predictions in multiple aspects. Firstly, contrasting to other models that require human-crafted features such as protein–protein interactions, protein kinase family classification, or sequence similarity (Kirchoff and Gomez, 2022; Luo *et al.*, 2019; Yang *et al.*, 2021), Phosformer utilizes a more unsupervised approach by allowing the Transformer architecture to generate its own features based solely on primary, unaligned sequences. This gives the model unique flexibility to accommodate and make predictions on any input kinases and substrate. In addition, recognizing the challenge of curation of the negative examples for kinase-specific phosphorylation prediction, we developed a novel approach to defining the negative examples by incorporating multi-level negative sampling in the training. This novel data sampling strategy helps to improve the model’s performance as well as reduce the false-positive prediction rate by a large margin. Apart from that, unlike the many previous models (Luo *et al.*, 2019; Yang *et al.*, 2021) that developed separate models for each kinase group or family, Phosformer unites for all kinases under a single architecture. The unified model allows Phosformer to maximize the sharing of knowledge between kinase families thus boosting the performance of less-represented families. Finally, different from most deep learning models that lack transparency, Phosformer is highly interpretable with its attention mechanism and our developed substrate attention visualization. The model is capable of translating divergent lengths of kinase-substrate inputs as fix-size embedding vectors so as to investigate the landscape of kinase-substrate specificity determinants. Further analysis of the trained model reveals that Phosformer can learn substrate specificity motifs (Fig. 7) and distinguish between functionally distinct protein kinase families—acidophilic, basophilic or proline-directed (Fig. 6B). This ability is corroborated by previous results which have shown that Transformer protein language models are unsupervised learners for biochemical (Elnaggar *et al.*, 2021; Rives *et al.*, 2021), structural (Rao *et al.*, 2020) and evolutionary features (Yeung *et al.*, 2023a, b).

Despite Phosformer’s promising performance, we recognize potential areas for future improvements. Due to computational resource limitations, the Phosformer model only takes the kinase domain sequence and substrate peptide as inputs. This inevitably assumes that kinase–substrate associations are confined to these sequence regions. Incorporating the full kinase and substrate sequences would presumably enhance prediction accuracy by accounting for other contextual information encoded in full-length sequences such as cellular localization and protein–protein interactions. In addition, defining negative examples for kinase-specific phosphosite prediction is also a major challenge. Although we partially circumvent this issue by multi-level negative sampling, training the model with an experimentally validated negative dataset would likely reduce FPRs and improve performance even further. Lastly, requiring only the arbitrary kinase and substrate pair as input, Phosformer can theoretically be used to predict kinase-substrate specificity beyond the training dataset. However, this may require an additional learning scheme to accommodate the model using zero-shot learning techniques which will be explored in future studies.

## Supplementary Material

btad046_Supplementary_Data

## Data avaliability

Code and data are publically available in a repository and can be accessed via https://github.com/esbgkannan/phosformer.
