# Supplementary material for: Phosformer: an explainable transformer model for protein kinase-specific phosphorylation predictions
Source: Bioinformatics. 2023 Jan 24;39(2):btad046. doi: 10.1093/bioinformatics/btad046 (PMC9900213; doi:10.1093/bioinformatics/btad046)

Supplementary Material:

**Phosformer: an interpretable Transformer model for kinase-specific phosphosite predictions**

Zhongliang Zhou^1, †^, Wayland Yeung^2, †^, Nathan Gravel^2^, Mariah Salcedo^2^, Saber Soleymani ^1^, Sheng Li^4,*^ and Natarajan Kannan^2, *^

1 School of Computing, University of Georgia, GA 30605, USA

2 Institute of Bioinformatics, University of Georgia, GA 30605, USA

3 Department of Biochemistry and Molecular Biology, University of Georgia, GA 30605, USA

4 School of Data Science, University of Virginia, VA 22903, USA

^†^ These authors contributed equally to the work,

^*^ To whom correspondence should be addressed

**Table S1.** The statistics of curated dataset for kinase-specific phosphorylation. The data are curated from four database and manually recheck for redundancy.

| Group | Family | counts |
| --- | --- | --- |
| Atypical | **Alpha** | 88 |
| Atypical | **FAM20** | 17 |
| Atypical | **PIKK** | 919 |
| Atypical | **PIPK** | 1 |
| Atypical | **ABC1** | 1 |
| Atypical | **Bud32** | 1 |
| Atypical | **RIO** | 3 |
| AGC | **Akt** | 718 |
| AGC | **DMPK** | 192 |
| AGC | **GRK** | 290 |
| AGC | **MAST** | 14 |
| AGC | **NDR** | 99 |
| AGC | **PDK1** | 82 |
| AGC | **PKA** | 1729 |
| AGC | **PKC** | 2098W |
| AGC | **PKG** | 205 |
| AGC | **PKN** | 45 |
| AGC | **RSK** | 519 |
| AGC | **RSKR** | 1 |
| AGC | **SGK** | 102 |
| CAMK | **CAMK1** | 112 |
| CAMK | **CAMK2** | 617 |
| CAMK | **CAMKL** | 921 |
| CAMK | **CASK** | 6 |
| CAMK | **DAPK** | 134 |
| CAMK | **DCAMKL** | 10 |
| CAMK | **MAPKAPK** | 219 |
| CAMK | **MLCK** | 43 |
| CAMK | **PHK** | 14 |
| CAMK | **PIM** | 177 |
| CAMK | **PKD** | 146 |
| CAMK | **RAD53** | 103 |
| CAMK | **STK33** | 5 |
| CAMK | **SgK495** | 10 |
| CAMK | **TSSK** | 8 |
| CAMK | **Trbl** | 16 |
| CAMK | **Trio** | 1 |
| CK1 | **CK1-A** | 205 |
| CK1 | **CK1-D** | 262 |
| CK1 | **CK1-G** | 22 |
| CK1 | **TTBK** | 21 |
| CK1 | **VRK** | 43 |
| CMGC | **CDK** | 2574 |
| CMGC | **CDKL** | 29 |
| CMGC | **CK2** | 1136 |
| CMGC | **CLK** | 122 |
| CMGC | **DYRK** | 350 |
| CMGC | **GSK** | 777 |
| CMGC | **MAPK** | 3126 |
| CMGC | **RCK** | 12 |
| CMGC | **SRPK** | 100 |
| NEK | **NEK1** | 52 |
| NEK | **NEK11** | 4 |
| NEK | **NEK2** | 45 |
| NEK | **NEK4** | 15 |
| NEK | **NEK6** | 42 |
| NEK | **NEK9** | 19 |
| Other | **Aur** | 448 |
| Other | **BUB** | 86 |
| Other | **CAMKK** | 43 |
| Other | **CDC7** | 50 |
| Other | **Haspin** | 4 |
| Other | **IKK** | 382 |
| Other | **IRE** | 1 |
| Other | **KIS** | 32 |
| Other | **MOS** | 11 |
| Other | **NAK** | 30 |
| Other | **NKF2** | 17 |
| Other | **NKF4** | 4 |
| Other | **NRBP** | 5 |
| Other | **PEK** | 56 |
| Other | **PLK** | 479 |
| Other | **SCY1** | 18 |
| Other | **SgK493** | 4 |
| Other | **Slob** | 8 |
| Other | **TLK** | 9 |
| Other | **TOPK** | 15 |
| Other | **TTK** | 70 |
| Other | **ULK** | 115 |
| Other | **VPS15** | 4 |
| Other | **WEE** | 82 |
| Other | **WNK** | 49 |
| RGC |  | 6 |
| STE | **COT** | 34 |
| STE | **NIK** | 24 |
| STE | **STE11** | 78 |
| STE | **STE20** | 611 |
| STE | **STE7** | 154 |
| TK | **ALK** | 17 |
| TK | **Abl** | 355 |
| TK | **Ack** | 12 |
| TK | **Axl** | 38 |
| TK | **Csk** | 29 |
| TK | **DDR** | 7 |
| TK | **EGFR** | 194 |
| TK | **Eph** | 66 |
| TK | **FAK** | 100 |
| TK | **FGFR** | 106 |
| TK | **Fer** | 43 |
| TK | **InsR** | 142 |
| TK | **Jak** | 163 |
| TK | **Lmr** | 4 |
| TK | **Met** | 75 |
| TK | **Musk** | 5 |
| TK | **PDGFR** | 101 |
| TK | **Ret** | 42 |
| TK | **STYK** | 6 |
| TK | **Src** | 1688 |
| TK | **Syk** | 169 |
| TK | **Tec** | 111 |
| TK | **Tie** | 9 |
| TK | **Trk** | 52 |
| TK | **VEGFR** | 26 |
| TKL | **IRAK** | 65 |
| TKL | **LIMK** | 15 |
| TKL | **LRRK** | 116 |
| TKL | **MLK** | 172 |
| TKL | **RAF** | 83 |
| TKL | **RIPK** | 54 |
| TKL | **STKR** | 98 |
| TKL | **TESK** | 15 |

**Table S2.** The detailed column definition of curated dataset.

| Kinase\|family | Kinase family information manually curated by literature comparison with three level hierarchy |
| --- | --- |
| Kinase\|uniprot | Kinase uniprot id |
| Kinase\|organisim | Organism where the kinase found |
| Kinase\|sequence | Sequence accessed from Uniprot database |
| Substrate\|uniprot | Substrate uniprot id |
| Substrate\|organism | Organism where the substrate found |
| Substrate\|sequence | 15-mer sequence of the substrate centered around the phosphorylation site |
| Substrate\|resnum | Residue number of the phosphorylation site |
| Substrate\|residue | Residue type of the phosphorylation site |
| Source\|PhosphoSitePlus | Appearances in PhosphoSitePlus |
| Source\|PhosphoELM | Appearances in PhosphoELM |
| Source\|PhosphoNetworks | Appearances in PhosphoNetworks |

**Figure S1.** The illustration of steps for calculating the defined substate attention values.


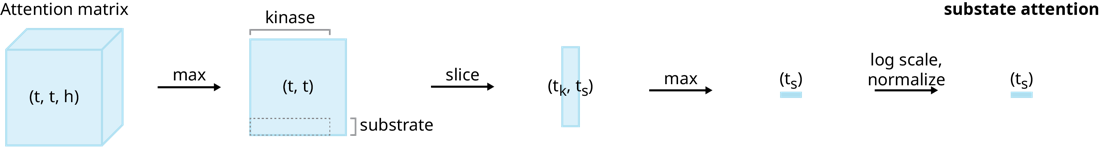


The intuition is to localize the contribution of each substrate residue to the kinase region across multiple attention heads. To calculate such scores, the last layer of the attention values is first extracted from the Phosformer encoder module. These attention values are of shape $\left( t,t,h \right)$, where the t corresponds to the length of the input tokens and the h is the number of attention heads. We first perform a max operation along the multiple attention head’s dimension which works similarly to the max pooling. This will reduce our attention matrix into a matrix of shape $\left( t,t \right)$. We then slice the substrate attentions from the reduced matrix to get the per residue attention vector of shape $(t_{k},t_{s})$, where k is the length of the kinase and s is the length of the substrate. After that, we perform another max operation along the kinase dimension and use log function together with normalization to get the final substrate attention of $\left( t_{s} \right)$. The final attention score has an identical length to the substrate so that it can be mapped directly to each amino acid.

**Table S3.** The AUC ROC, AUC PRC and FPR scores for various Phosformer models on the original testing dataset.

|  | **w/ pretrain** | **w/ augments** | **use easy negative samples** | **use hard negative**  **samples** | **w/ focal loss** | **pos/neg ratio** | **AUC ROC** | **AUC PRC** | **FPR** |
| --- | --- | --- | --- | --- | --- | --- | --- | --- | --- |
| **Model 1** |  |  |  | **X** |  | 1:(1+0) | 0.577 | 0.580 | 0.038 |
| **Model 2** | **X** |  |  | **X** |  | 1:(1+0) | 0.883 | 0.861 | 0.224 |
| **Model 3** | **X** | **X** |  | **X** |  | 1:(1+0) | 0.883 | 0.867 | 0.161 |
| **Model 4** | **X** | **X** | **X** |  |  | 1:(0+1) | 0.880 | 0.862 | 0.103 |
| **Model 5** | **X** | **X** | **X** | **X** | **X** | 1:(1+1) | 0.885 | 0.873 | 0.070 |
| **Model 6** | **X** | **X** | **X** | **X** |  | 1:(8+8) | 0.865 | 0.839 | 0.024 |
| **Model 7** | **X** | **X** | **X** | **X** | **X** | **1:(8+8)** | **0.885** | **0.877** | **0.023** |

In addition to the four models reported in the paper, we added 3 extra experiments. The first experiment used a randomly initialized model and fine-tuned with the kinase-specific phosphorylation dataset which is denoted as Model 1 in the above table. Comparing Model 1 with Model 2, a randomly initialized model struggled to converge under the same settings, which further emphasizes the necessity of using a pretrained language model. The second experiment used only the non-phosphosites as the negative dataset with a positive-to-negative ratio of 1 to 1 which is denoted as Model 4. Compared to using the non-specific phosphosites as the negative sites which is Model 3, this model yields a slightly better AUC ROC score and a worse AUC PRC score. In light of this finding, we argue that using a combination of non-phosphosites and non-specific phosphosites can complement each other and thus provide a better training dataset which can be seen from Model 5 result. The third experiment tested the impact of the focal loss under an extreme positive-to-negative ratio setting shown as Model 6. Compared to same positive-to-negative ratio of 1 to 16 using focal loss as Model 7, it can be noticed that using focal loss helps to handle the data imbalance issue thus providing performance improvement shown from both AUC ROC and AUC PRC scores.

**Figure S2.** The AUC ROC and AUC PRC Plot for various Phosformer models on the original testing dataset.


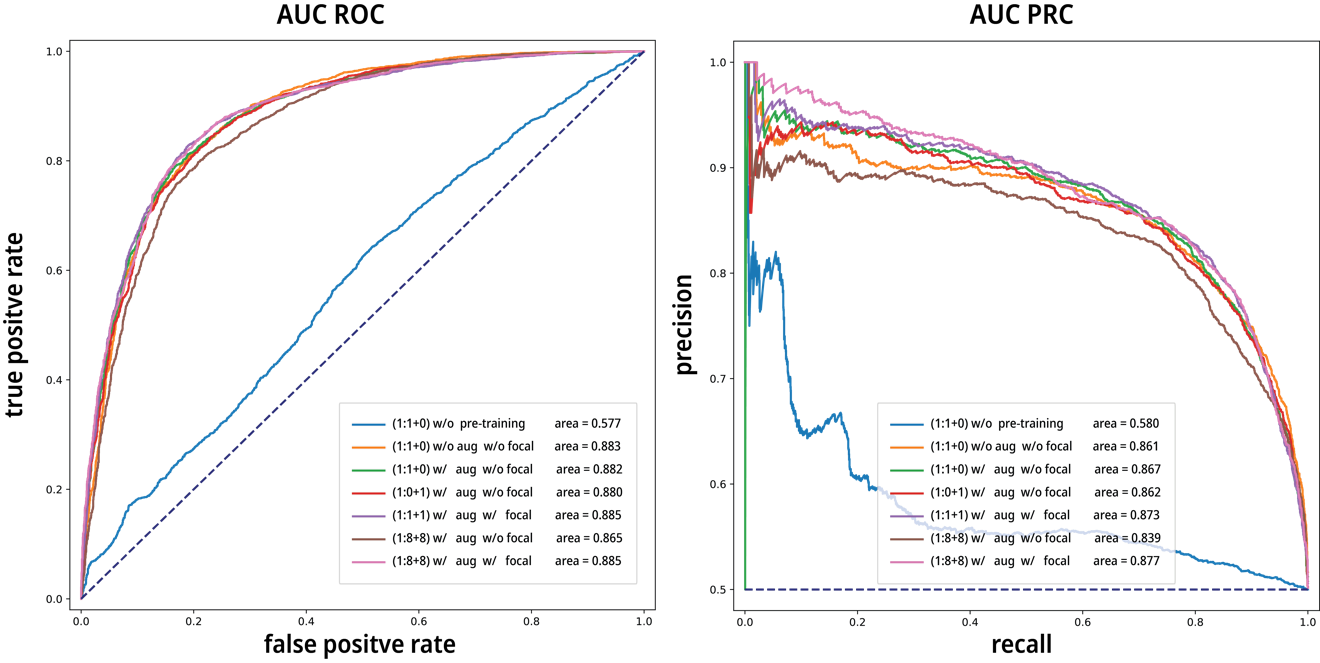


**Figure S3.** The AUC ROC and AUC PRC Plot for various Phosformer models on the augmented testing dataset.


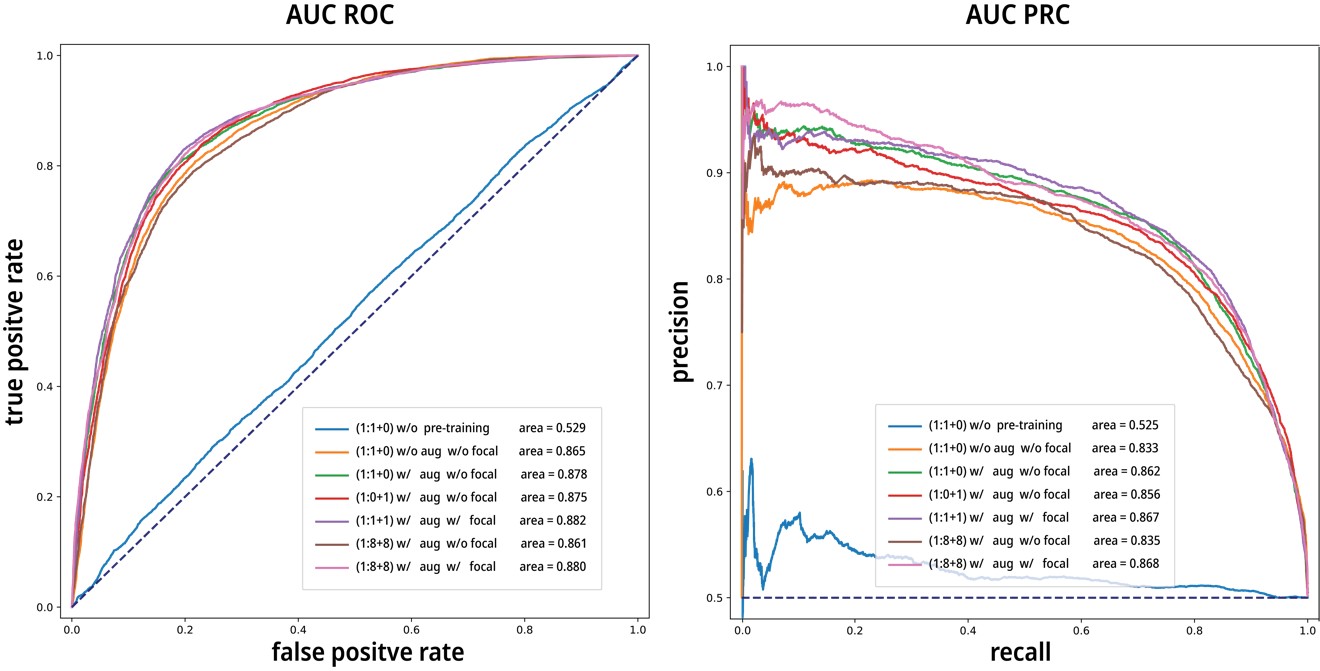


The augmented testing dataset is derived from the original testing dataset with shifted kinase domain by up to 3 positions. This will help evaluate whether the model is robust to permutations and whether the model is overfitting by simply recognizing kinase patterns. Comparing the first two models with a positive-to-negative ratio of 1 to 1, it can be seen that applying augmentations significantly boosted the performance of both AUC ROC and AUC PRC.

**Table S4.** The detailed AUC and AUC PRC scores for the best performing Phosformer model on the original testing dataset.

| **Group** | **Family** | **Gene** | **Kinase** | **AUC PRC** | **AUC ROC** | **Accuracy** | **MCC** | **F1** |
| --- | --- | --- | --- | --- | --- | --- | --- | --- |
| **Total** |  |  |  | 0.877 | 0.885 | 0.688 | 0.450 | 0.662 |
| **Atypical** | **Alpha** |  |  | 0.740 | 0.716 | 0.500 | 0.000 | 0.333 |
| **Atypical** | **Alpha** | **TRPM7** | **Q96QT4** | 0.740 | 0.716 | 0.500 | 0.000 | 0.333 |
| **Atypical** | **PIKK** |  |  | 0.864 | 0.849 | 0.733 | 0.533 | 0.716 |
| **Atypical** | **PIKK** | **ATM** | **Q13315** | 0.938 | 0.939 | 0.880 | 0.770 | 0.880 |
| **Atypical** | **PIKK** | **ATR** | **Q13535** | 0.889 | 0.892 | 0.833 | 0.684 | 0.831 |
| **Atypical** | **PIKK** | **MTOR** | **P42345** | 0.563 | 0.562 | 0.524 | 0.156 | 0.384 |
| **Atypical** | **PIKK** | **MTOR** | **Q9JLN9** | 0.636 | 0.587 | 0.500 | 0.000 | 0.333 |
| **Atypical** | **PIKK** | **PRKDC** | **P78527** | 0.967 | 0.959 | 0.786 | 0.632 | 0.775 |
| **AGC** |  |  |  | 0.900 | 0.898 | 0.748 | 0.549 | 0.736 |
| **AGC** | **Akt** |  |  | 0.896 | 0.901 | 0.755 | 0.552 | 0.746 |
| **AGC** | **Akt** | **AKT1** | **P31749** | 0.917 | 0.916 | 0.785 | 0.607 | 0.778 |
| **AGC** | **Akt** | **AKT1** | **P31750** | 0.771 | 0.844 | 0.688 | 0.378 | 0.686 |
| **AGC** | **Akt** | **AKT2** | **P31751** | 0.884 | 0.840 | 0.625 | 0.378 | 0.564 |
| **AGC** | **DMPK** |  |  | 0.961 | 0.959 | 0.773 | 0.612 | 0.760 |
| **AGC** | **DMPK** | **ROCK1** | **Q13464** | 0.961 | 0.959 | 0.773 | 0.612 | 0.760 |
| **AGC** | **GRK** |  |  | 0.740 | 0.707 | 0.563 | 0.258 | 0.459 |
| **AGC** | **GRK** | **ARBK1** | **P25098** | 0.740 | 0.707 | 0.563 | 0.258 | 0.459 |
| **AGC** | **PDK1** |  |  | 0.902 | 0.906 | 0.750 | 0.577 | 0.733 |
| **AGC** | **PDK1** | **PDPK1** | **O15530** | 0.902 | 0.906 | 0.750 | 0.577 | 0.733 |
| **AGC** | **PKA** |  |  | 0.903 | 0.907 | 0.776 | 0.592 | 0.768 |
| **AGC** | **PKA** | **KAPCA** | **P00517** | 0.866 | 0.881 | 0.683 | 0.423 | 0.662 |
| **AGC** | **PKA** | **KAPCA** | **P17612** | 0.883 | 0.889 | 0.772 | 0.583 | 0.764 |
| **AGC** | **PKA** | **KAPCA** | **P05132** | 0.968 | 0.963 | 0.833 | 0.692 | 0.830 |
| **AGC** | **PKA** | **KAPCA** | **P27791** | 0.976 | 0.975 | 0.857 | 0.745 | 0.854 |
| **AGC** | **PKC** |  |  | 0.894 | 0.884 | 0.717 | 0.503 | 0.698 |
| **AGC** | **PKC** | **KPCA** | **P17252** | 0.925 | 0.923 | 0.731 | 0.528 | 0.715 |
| **AGC** | **PKC** | **KPCA** | **P20444** | 0.863 | 0.861 | 0.625 | 0.308 | 0.590 |
| **AGC** | **PKC** | **KPCA** | **P05696** | 0.853 | 0.866 | 0.680 | 0.469 | 0.643 |
| **AGC** | **PKC** | **KPCB** | **P05771** | 0.948 | 0.948 | 0.825 | 0.694 | 0.819 |
| **AGC** | **PKC** | **KPCD** | **Q05655** | 0.838 | 0.849 | 0.650 | 0.364 | 0.619 |
| **AGC** | **PKC** | **KPCE** | **Q02156** | 0.861 | 0.863 | 0.750 | 0.539 | 0.741 |
| **AGC** | **PKC** | **KPCG** | **P05129** | 1.000 | 1.000 | 1.000 | 1.000 | 1.000 |
| **AGC** | **PKC** | **KPCI** | **P41743** | 0.782 | 0.688 | 0.688 | 0.480 | 0.654 |
| **AGC** | **PKC** | **KPCT** | **Q04759** | 0.927 | 0.906 | 0.750 | 0.577 | 0.733 |
| **AGC** | **PKC** | **KPCZ** | **Q05513** | 0.776 | 0.745 | 0.609 | 0.303 | 0.555 |
| **AGC** | **PKG** |  |  | 0.955 | 0.947 | 0.808 | 0.632 | 0.805 |
| **AGC** | **PKG** | **KGP1** | **Q13976** | 0.955 | 0.947 | 0.808 | 0.632 | 0.805 |
| **AGC** | **RSK** |  |  | 0.929 | 0.944 | 0.775 | 0.587 | 0.768 |
| **AGC** | **RSK** | **KS6A1** | **Q15418** | 0.971 | 0.961 | 0.719 | 0.529 | 0.695 |
| **AGC** | **RSK** | **KS6A3** | **P51812** | 0.863 | 0.864 | 0.889 | 0.778 | 0.889 |
| **AGC** | **RSK** | **KS6B1** | **P23443** | 0.922 | 0.951 | 0.767 | 0.566 | 0.760 |
| **AGC** | **SGK** |  |  | 0.979 | 0.975 | 0.944 | 0.894 | 0.944 |
| **AGC** | **SGK** | **SGK1** | **O00141** | 0.979 | 0.975 | 0.944 | 0.894 | 0.944 |
| **CAMK** |  |  |  | 0.813 | 0.830 | 0.667 | 0.392 | 0.643 |
| **CAMK** | **CAMK2** |  |  | 0.817 | 0.826 | 0.682 | 0.421 | 0.661 |
| **CAMK** | **CAMK2** | **KCC2A** | **Q9UQM7** | 0.847 | 0.844 | 0.686 | 0.457 | 0.657 |
| **CAMK** | **CAMK2** | **KCC2A** | **P11798** | 0.699 | 0.741 | 0.611 | 0.236 | 0.600 |
| **CAMK** | **CAMK2** | **KCC2A** | **P11275** | 0.786 | 0.818 | 0.667 | 0.364 | 0.653 |
| **CAMK** | **CAMK2** | **KCC2B** | **P08413** | 0.966 | 0.959 | 0.786 | 0.632 | 0.775 |
| **CAMK** | **CAMKL** |  |  | 0.828 | 0.824 | 0.684 | 0.430 | 0.661 |
| **CAMK** | **CAMKL** | **AAPK1** | **Q13131** | 0.881 | 0.882 | 0.734 | 0.499 | 0.726 |
| **CAMK** | **CAMKL** | **AAPK1** | **Q5EG47** | 0.966 | 0.959 | 0.857 | 0.745 | 0.854 |
| **CAMK** | **CAMKL** | **AAPK2** | **P54646** | 0.681 | 0.661 | 0.591 | 0.204 | 0.569 |
| **CAMK** | **CAMKL** | **CHK1** | **O14757** | 0.847 | 0.797 | 0.633 | 0.392 | 0.576 |
| **CAMK** | **CAMKL** | **STK11** | **Q15831** | 0.758 | 0.694 | 0.643 | 0.408 | 0.591 |
| **CAMK** | **DAPK** |  |  | 0.949 | 0.953 | 0.500 | 0.000 | 0.333 |
| **CAMK** | **DAPK** | **ST17A** | **Q9UEE5** | 0.949 | 0.953 | 0.500 | 0.000 | 0.333 |
| **CAMK** | **MAPKAPK** |  |  | 1.000 | 1.000 | 0.767 | 0.603 | 0.753 |
| **CAMK** | **MAPKAPK** | **MAPK2** | **P49137** | 1.000 | 1.000 | 0.767 | 0.603 | 0.753 |
| **CAMK** | **PIM** |  |  | 0.755 | 0.789 | 0.619 | 0.258 | 0.605 |
| **CAMK** | **PIM** | **PIM1** | **P11309** | 0.851 | 0.864 | 0.654 | 0.333 | 0.641 |
| **CAMK** | **PIM** | **PIM2** | **Q9P1W9** | 0.497 | 0.594 | 0.563 | 0.135 | 0.547 |
| **CAMK** | **PKD** |  |  | 0.635 | 0.751 | 0.692 | 0.404 | 0.685 |
| **CAMK** | **PKD** | **KPCD1** | **Q15139** | 0.635 | 0.751 | 0.692 | 0.404 | 0.685 |
| **CAMK** | **RAD53** |  |  | 0.699 | 0.720 | 0.533 | 0.111 | 0.444 |
| **CAMK** | **RAD53** | **CHK2** | **O96017** | 0.699 | 0.720 | 0.533 | 0.111 | 0.444 |
| **CK1** |  |  |  | 0.822 | 0.808 | 0.649 | 0.400 | 0.605 |
| **CK1** | **CK1-A** |  |  | 0.825 | 0.818 | 0.667 | 0.414 | 0.635 |
| **CK1** | **CK1-A** | **KC1A** | **P48729** | 0.825 | 0.818 | 0.667 | 0.414 | 0.635 |
| **CK1** | **CK1-D** |  |  | 0.827 | 0.806 | 0.633 | 0.392 | 0.576 |
| **CK1** | **CK1-D** | **KC1D** | **P48730** | 0.833 | 0.817 | 0.618 | 0.365 | 0.552 |
| **CK1** | **CK1-D** | **KC1E** | **P49674** | 0.800 | 0.769 | 0.654 | 0.426 | 0.607 |
| **CMGC** |  |  |  | 0.882 | 0.898 | 0.643 | 0.383 | 0.598 |
| **CMGC** | **CDK** |  |  | 0.884 | 0.907 | 0.580 | 0.280 | 0.494 |
| **CMGC** | **CDK** | **CDK1** | **P06493** | 0.905 | 0.902 | 0.572 | 0.279 | 0.476 |
| **CMGC** | **CDK** | **CDK2** | **P24941** | 0.871 | 0.902 | 0.630 | 0.387 | 0.572 |
| **CMGC** | **CDK** | **CDK4** | **P11802** | 0.856 | 0.913 | 0.553 | 0.171 | 0.470 |
| **CMGC** | **CDK** | **CDK5** | **Q00535** | 0.873 | 0.907 | 0.578 | 0.264 | 0.497 |
| **CMGC** | **CDK** | **CDK5** | **P49615** | 0.877 | 0.931 | 0.583 | 0.302 | 0.496 |
| **CMGC** | **CDK** | **CDK6** | **Q00534** | 1.000 | 1.000 | 0.556 | 0.243 | 0.446 |
| **CMGC** | **CDK** | **CDK7** | **P50613** | 0.819 | 0.792 | 0.563 | 0.258 | 0.459 |
| **CMGC** | **CDK** | **CDK9** | **P50750** | 0.939 | 0.930 | 0.500 | 0.000 | 0.333 |
| **CMGC** | **CK2** |  |  | 0.884 | 0.881 | 0.769 | 0.571 | 0.763 |
| **CMGC** | **CK2** | **CSK21** | **P68400** | 0.880 | 0.879 | 0.763 | 0.559 | 0.756 |
| **CMGC** | **CK2** | **CSK21** | **Q60737** | 0.937 | 0.901 | 0.889 | 0.798 | 0.888 |
| **CMGC** | **CK2** | **CSK21** | **P19139** | 0.891 | 0.870 | 0.750 | 0.524 | 0.744 |
| **CMGC** | **CLK** |  |  | 0.842 | 0.781 | 0.625 | 0.378 | 0.564 |
| **CMGC** | **CLK** | **CLK2** | **P49760** | 0.842 | 0.781 | 0.625 | 0.378 | 0.564 |
| **CMGC** | **DYRK** |  |  | 0.737 | 0.731 | 0.500 | 0.000 | 0.333 |
| **CMGC** | **DYRK** | **DYRK2** | **Q92630** | 0.697 | 0.610 | 0.500 | 0.000 | 0.333 |
| **CMGC** | **DYRK** | **HIPK2** | **Q9H2X6** | 0.840 | 0.864 | 0.500 | 0.000 | 0.333 |
| **CMGC** | **GSK** |  |  | 0.809 | 0.810 | 0.615 | 0.318 | 0.563 |
| **CMGC** | **GSK** | **GSK3A** | **P49840** | 0.819 | 0.855 | 0.656 | 0.378 | 0.627 |
| **CMGC** | **GSK** | **GSK3B** | **P49841** | 0.791 | 0.791 | 0.595 | 0.277 | 0.532 |
| **CMGC** | **GSK** | **GSK3B** | **Q9WV60** | 0.829 | 0.790 | 0.700 | 0.500 | 0.670 |
| **CMGC** | **MAPK** |  |  | 0.915 | 0.924 | 0.657 | 0.414 | 0.616 |
| **CMGC** | **MAPK** | **MK01** | **P28482** | 0.928 | 0.948 | 0.660 | 0.402 | 0.626 |
| **CMGC** | **MAPK** | **MK01** | **P63085** | 0.969 | 0.963 | 0.817 | 0.681 | 0.810 |
| **CMGC** | **MAPK** | **MK03** | **P27361** | 0.885 | 0.908 | 0.646 | 0.380 | 0.606 |
| **CMGC** | **MAPK** | **MK03** | **Q63844** | 0.960 | 0.959 | 0.818 | 0.683 | 0.812 |
| **CMGC** | **MAPK** | **MK03** | **P21708** | 1.000 | 1.000 | 0.786 | 0.632 | 0.775 |
| **CMGC** | **MAPK** | **MK07** | **Q13164** | 0.741 | 0.633 | 0.500 | 0.000 | 0.333 |
| **CMGC** | **MAPK** | **MK08** | **P45983** | 0.925 | 0.929 | 0.627 | 0.382 | 0.567 |
| **CMGC** | **MAPK** | **MK09** | **P45984** | 0.966 | 0.972 | 0.708 | 0.513 | 0.681 |
| **CMGC** | **MAPK** | **MK10** | **P53779** | 0.983 | 0.983 | 0.682 | 0.471 | 0.646 |
| **CMGC** | **MAPK** | **MK11** | **Q15759** | 0.877 | 0.816 | 0.500 | 0.000 | 0.333 |
| **CMGC** | **MAPK** | **MK14** | **Q16539** | 0.838 | 0.856 | 0.556 | 0.243 | 0.446 |
| **CMGC** | **MAPK** | **MK14** | **P47811** | 0.916 | 0.920 | 0.650 | 0.420 | 0.601 |
| **CMGC** | **SRPK** |  |  | 0.944 | 0.938 | 0.813 | 0.674 | 0.806 |
| **CMGC** | **SRPK** | **SRPK2** | **P78362** | 0.944 | 0.938 | 0.813 | 0.674 | 0.806 |
| **Other** |  |  |  | 0.820 | 0.806 | 0.588 | 0.303 | 0.506 |
| **Other** | **Aur** |  |  | 0.891 | 0.871 | 0.645 | 0.413 | 0.594 |
| **Other** | **Aur** | **AURKA** | **O14965** | 0.840 | 0.866 | 0.571 | 0.277 | 0.475 |
| **Other** | **Aur** | **AURKB** | **Q96GD4** | 0.912 | 0.878 | 0.691 | 0.486 | 0.659 |
| **Other** | **BUB** |  |  | 0.854 | 0.813 | 0.708 | 0.458 | 0.695 |
| **Other** | **BUB** | **BUB1** | **O43683** | 0.854 | 0.813 | 0.708 | 0.458 | 0.695 |
| **Other** | **IKK** |  |  | 0.833 | 0.832 | 0.609 | 0.349 | 0.538 |
| **Other** | **IKK** | **IKKA** | **O15111** | 0.774 | 0.766 | 0.500 | 0.000 | 0.333 |
| **Other** | **IKK** | **IKKB** | **O14920** | 0.784 | 0.821 | 0.571 | 0.277 | 0.475 |
| **Other** | **IKK** | **IKKE** | **Q14164** | 0.944 | 0.939 | 0.571 | 0.277 | 0.475 |
| **Other** | **IKK** | **TBK1** | **Q9UHD2** | 0.875 | 0.848 | 0.706 | 0.509 | 0.678 |
| **Other** | **PLK** |  |  | 0.731 | 0.692 | 0.529 | 0.172 | 0.394 |
| **Other** | **PLK** | **PLK1** | **P53350** | 0.731 | 0.692 | 0.529 | 0.172 | 0.394 |
| **Other** | **TTK** |  |  | 0.954 | 0.950 | 0.500 | 0.000 | 0.333 |
| **Other** | **TTK** | **TTK** | **P33981** | 0.954 | 0.950 | 0.500 | 0.000 | 0.333 |
| **Other** | **ULK** |  |  | 0.838 | 0.840 | 0.500 | 0.000 | 0.333 |
| **Other** | **ULK** | **ULK1** | **O75385** | 0.838 | 0.840 | 0.500 | 0.000 | 0.333 |
| **Other** | **WEE** |  |  | 0.874 | 0.876 | 0.545 | 0.218 | 0.427 |
| **Other** | **WEE** | **WEE1** | **P30291** | 0.874 | 0.876 | 0.545 | 0.218 | 0.427 |
| **STE** |  |  |  | 0.844 | 0.828 | 0.716 | 0.487 | 0.700 |
| **STE** | **STE20** |  |  | 0.844 | 0.828 | 0.716 | 0.487 | 0.700 |
| **STE** | **STE20** | **PAK1** | **Q13153** | 0.850 | 0.810 | 0.738 | 0.505 | 0.731 |
| **STE** | **STE20** | **PAK2** | **Q13177** | 0.738 | 0.813 | 0.500 | 0.000 | 0.333 |
| **STE** | **STE20** | **PAK4** | **O96013** | 0.956 | 0.938 | 0.875 | 0.775 | 0.873 |
| **TK** |  |  |  | 0.910 | 0.931 | 0.789 | 0.600 | 0.785 |
| **TK** | **Abl** |  |  | 0.961 | 0.966 | 0.808 | 0.641 | 0.804 |
| **TK** | **Abl** | **ABL1** | **P00519** | 0.961 | 0.966 | 0.808 | 0.641 | 0.804 |
| **TK** | **EGFR** |  |  | 0.883 | 0.925 | 0.737 | 0.522 | 0.725 |
| **TK** | **EGFR** | **EGFR** | **P00533** | 0.883 | 0.925 | 0.737 | 0.522 | 0.725 |
| **TK** | **FGFR** |  |  | 0.850 | 0.891 | 0.875 | 0.750 | 0.875 |
| **TK** | **FGFR** | **FGFR1** | **P11362** | 0.850 | 0.891 | 0.875 | 0.750 | 0.875 |
| **TK** | **InsR** |  |  | 0.951 | 0.938 | 0.833 | 0.671 | 0.833 |
| **TK** | **InsR** | **INSR** | **P06213** | 0.951 | 0.938 | 0.833 | 0.671 | 0.833 |
| **TK** | **Jak** |  |  | 0.844 | 0.859 | 0.625 | 0.258 | 0.619 |
| **TK** | **Jak** | **JAK2** | **O60674** | 0.844 | 0.859 | 0.625 | 0.258 | 0.619 |
| **TK** | **Src** |  |  | 0.907 | 0.927 | 0.780 | 0.584 | 0.775 |
| **TK** | **Src** | **FYN** | **P06241** | 0.897 | 0.916 | 0.803 | 0.620 | 0.801 |
| **TK** | **Src** | **LCK** | **P06239** | 0.947 | 0.950 | 0.789 | 0.610 | 0.784 |
| **TK** | **Src** | **LYN** | **P07948** | 0.987 | 0.988 | 0.850 | 0.734 | 0.847 |
| **TK** | **Src** | **SRC** | **P12931** | 0.895 | 0.919 | 0.765 | 0.557 | 0.759 |
| **TK** | **Src** | **SRC** | **P05480** | 0.804 | 0.878 | 0.714 | 0.447 | 0.708 |
| **TK** | **Syk** |  |  | 0.935 | 0.953 | 0.962 | 0.926 | 0.961 |
| **TK** | **Syk** | **KSYK** | **P43405** | 0.935 | 0.953 | 0.962 | 0.926 | 0.961 |
| **TKL** |  |  |  | 0.612 | 0.605 | 0.543 | 0.154 | 0.450 |
| **TKL** | **LRRK** |  |  | 0.504 | 0.462 | 0.538 | 0.200 | 0.414 |
| **TKL** | **LRRK** | **LRRK2** | **Q5S007** | 0.504 | 0.462 | 0.538 | 0.200 | 0.414 |
| **TKL** | **MLK** |  |  | 0.661 | 0.670 | 0.550 | 0.140 | 0.487 |
| **TKL** | **MLK** | **M3K7** | **O43318** | 0.661 | 0.670 | 0.550 | 0.140 | 0.487 |

**Figure S4. (A)** Two trees of the human protein kinome show the diversity of kinases upon which Phosformer can predict. T green branch indicates the model can make inferences about certain individuals. Black dots denote the model can only make family or group-level predictions.Recent models include Musitedeep, PhosIDN, DeepPhos, EMBER. (B) A histogram graph shows the number of predicted substrates for each protein human kinase family. (C) A log-scaled histogram graph shows the number of predicted kinases for each phosphorylate site. The x-axis is the number of predicted kinases per site and the y-axis is the count of sites that have the same number of kinase predictions. Overall, there are 80,344 sites that have more than one predicted kinase associations.


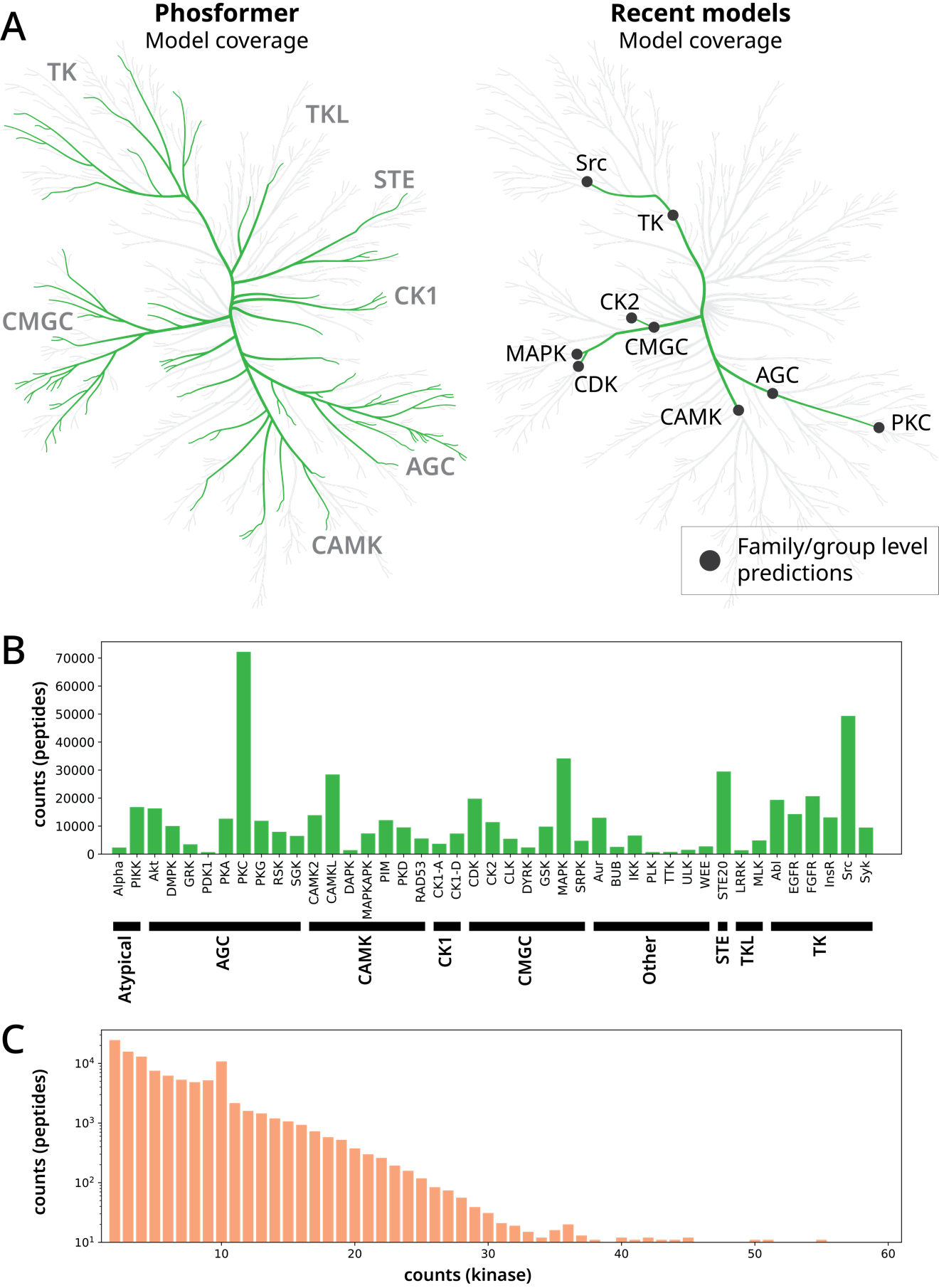


**Figure S5.** Sequence logos show predictions for diverse protein kinase families across the human proteome using Phosformer. The prediction is generated using the best-performing Phosformer model with the threshold set as 0.5. The kinase list contains 83 human kinases that were included in the testing set. The human phosphosites have 219,799 sites that are extracted from the curated database. Each kinase is annotated with a legend denoting its Uniprot ID, gene name, group hierarchy, and family hierarchy.


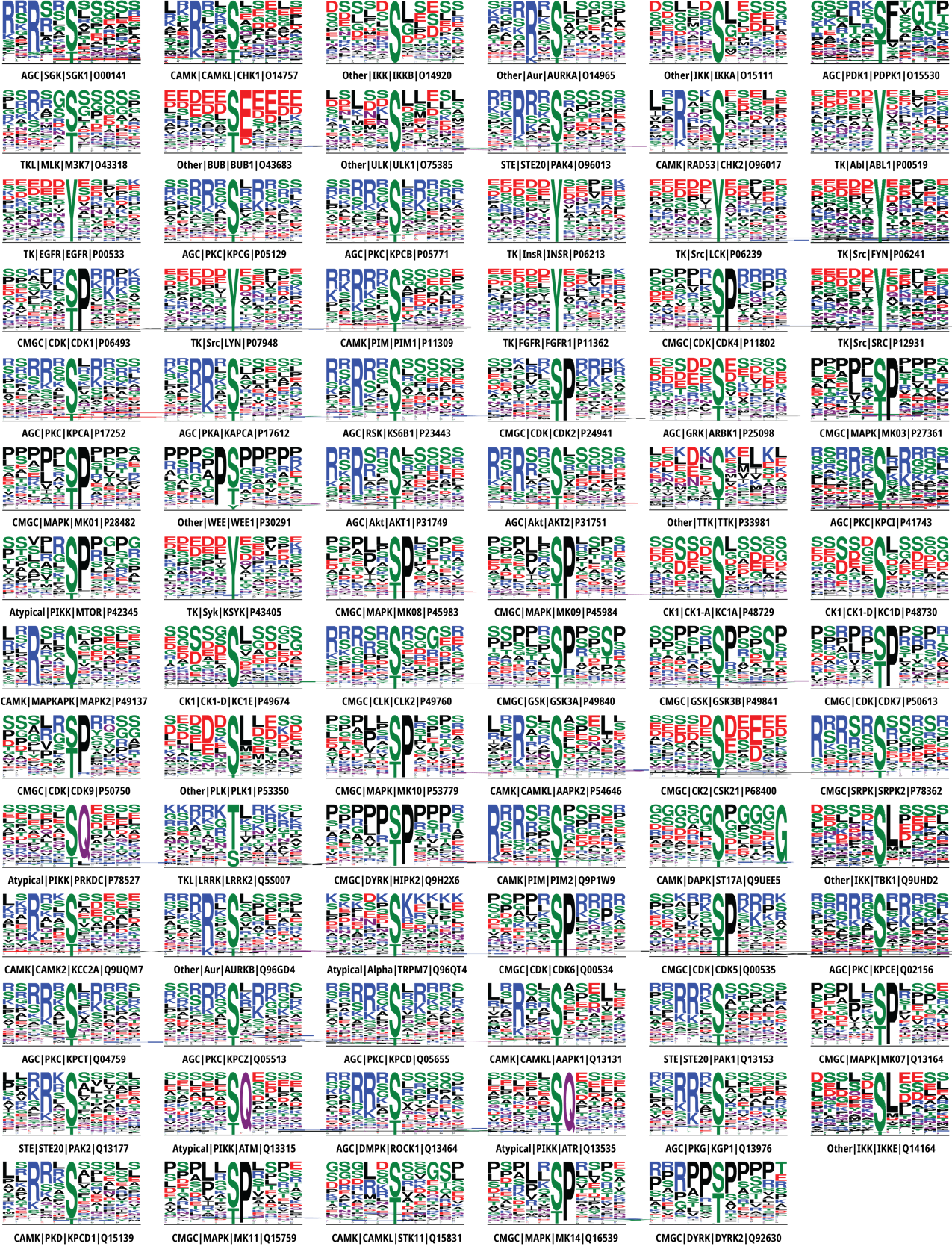

Supplement: btad046_Supplementary_Data [file btad046_supplementary_data.docx]
